# Genetic Variants Associated with Sensitive Skin: A Genome-Wide Association Study in Korean Women

**DOI:** 10.3390/life14111352

**Published:** 2024-10-22

**Authors:** Seoyoung Kim, Kyung-Won Hong, Mihyun Oh, Susun An, Jieun Han, Sodam Park, Goun Kim, Jae Youl Cho

**Affiliations:** 1 Safety and Microbiology Laboratory, Amorepacific R&I Center, Yongin 17074, Republic of Korea; ksy1414@amorepacific.com (S.K.); vine01@amorepacific.com (M.O.); ssan@amorepacific.com (S.A.); hje622@amorepacific.com (J.H.); sd-park@amorepacific.com (S.P.); kgu3423@amorepacific.com (G.K.); 2 Department of Integrative Biotechnology, Sungkyunkwan University, Suwon 16419, Republic of Korea; 3 Institute of Advanced Technology, Theragen Health Co., Ltd., Seongnam 13493, Republic of Korea; kyungwon.hong@theragenhealth.com

**Keywords:** sensitive skin, genome-wide association study, single-nucleotide polymorphism, *RASSF8*, *GPX1*

## Abstract

Sensitive skin (SS) is associated with discomfort, including burning, stinging, and itching. These symptoms are often exacerbated by environmental factors and personal care products. In this genome-wide association study (GWAS), we aimed to identify the genetic variants associated with SS in 1690 Korean female participants; 389 and 1301 participants exhibited sensitive and non-sensitive skin, respectively. Using a combination of self-reported questionnaires, patch tests, and sting tests, we selected 115 sensitive and 181 non-sensitive participants for genetic analysis. A GWAS was performed to identify the loci associated with SS. Although none of the single-nucleotide polymorphisms (SNPs) met the genome-wide significance threshold, we identified several SNPs with suggestive associations. SNP rs11689992 in the 2q11.3 region increased SS risk by approximately 3.67 times. SNP rs7614738 in the *USP4* locus elevated SS risk by 2.34 times and was found to be an expression quantitative trait locus for *GPX1*, a gene involved in oxidative stress and inflammation. Additionally, SNPs rs12306124 in the *RASSF8* locus and rs10483893 in the *NRXN3* region were identified. These results suggest that the genetic variations affecting oxidative stress, cell growth regulation, and neurobiology potentially influence skin sensitivity, providing a basis for further investigation and the development of personalized approaches to manage sensitive skin.

## 1. Introduction

Sensitive skin (SS) is associated with symptoms, such as burning, stinging, and itching, leading to subjective and objective discomfort in response to various stimuli including cosmetics, climate, and environmental factors [1,2,3]. Despite numerous studies, the pathophysiology of SS remains unclear owing to the interplays between various endogenous and exogenous factors [4]. SS is associated with a lower density of epidermal nerve fibers [5,6] and thinner stratum corneum [7], which can exacerbate skin sensitivity. Although SS is not classified as a dermatological disease, it can significantly impact the quality of life (QOL) [3,8].

Genome-wide association studies (GWAS) have identified several genetic variants associated with complex traits [9]. Recently, numerous skin-related genetic susceptibility factors, including age spots, freckles, double eyelids, and hair characteristics, have been reported [10]. Additionally, the genetic variations linked to skin phenotypes, such as acne and aging, have been reported in the Han Chinese populations [11,12]. Studies on atopic dermatitis in East Asia have provided important insights into the genetic basis of skin sensitivity [13,14,15,16]. However, further research on the correlations between SS and genetic variations is required.

In this study, we aimed to explore the genetic characteristics associated with SS by conducting a GWAS in Korean female patients. We focused on sensitive skin as defined by a diagnostic questionnaire, a 5% lactic acid stinging test (LAST) [17], and a 0.3% sodium lauryl sulfate (SLS) patch test [18]. We identified the novel genetic loci associated with SS that may contribute to personalized approaches for the management of sensitive skin.

## 2. Materials and Methods

### 2.1. Participants

We recruited 1690 female participants at the DERMAPRO Ltd. (Seoul, Republic of Korea), between June and July 2019. The study population comprised individuals living in the Seoul area, all sharing the same geographical and demographic background. Participants completed a self-report questionnaire covering general skin status, cosmetic usage, innate skin characteristics, environmental effects on skin, and personal habits (Appendix A). They also underwent a 0.3% SLS patch test and a 5% lactic acid stinging test. We selected 296 individuals, including 181 non-sensitive (NS) and 115 sensitive participants, for GWAS. This study adhered to the Declaration of Helsinki and was approved by the Amorepacific Institutional Review Board (IRB approval no. 2018-1SR-N063R). Written informed consent was obtained from all participants.

### 2.2. Sensitive and Non-Sensitive Skin Classification

In this study, to classify SS and NS, we initially used a sensitive skin questionnaire. Subsequently, to ensure accuracy in the classification of SS and NS, we conducted additional patch and sting tests. Among the questionnaire items (Appendix A), each of the five factors (Thickness, Skin Change, Cosmetic Issues, Skin Allergies, and Inflammation) associated with the general skin status was scored using a 1–3 scoring scale, and the sum of the five items’ scores was considered the “Category A” score. The remaining questionnaire items were scored as follows: 0 for “No” and 1 for “Yes” for each item, and the sum of the remaining items scores was considered the “Category B” score. Finally, the sensitive skin score was calculated by multiplying the Category A scores by 2 and the Category B scores by 3 and summing the results. Sensitive skin scores < 10 were classified as questionnaire NS, whereas sensitive skin scores ≥ 10 were classified as questionnaire SS (Supplementary Table S1).

#### 2.2.1. Patch Test (Skin Irritation Test)

For this test, 20 µL of 0.3% SLS (Sigma-Aldrich Co., St. Louis, MO, USA) was applied to an IQ chamber (Chemotechnique Diagnostics, Vellinge, Sweden) placed on the back of each participant for 24 h. Primary cutaneous irritation was then assessed using visual scoring based on a numerical erythema scale ranging from 0 to 4, allowing for the quantification of cutaneous irritation at 30 min and 1 d after removing the patch.

#### 2.2.2. Sting Test (Skin Sensory Test)

Participants rested for 10 min in a controlled environment (24 °C ± 4 °C, 40–45% relative humidity) after cleansing their face with water. 5% lactic acid (Sigma-Aldrich Co., St. Louis, MO, USA) in distilled water was then applied to the nasolabial fold and cheek; distilled water was used as the negative control. Stinging and burning reactions were recorded every 10 s and every minute for 9 min on a scale of 0 (none)–3 (severe).

### 2.3. SNP Microarray Genotyping

Buccal swab samples were collected using TheraKit (Theragen Bio Co., Ltd., Seongnam, Republic of Korea). DNA was extracted from the buccal swab samples using Exgene^TM^ Tissue SV (GeneAll, Seoul, Republic of Korea), amplified, and fragmented into 25–125 bp pieces. The fragments were purified, resuspended, and hybridized with the Theragen Precision Medicine Research Array (Theragen PMRA array) [19], a customized platform based on the Asia Precision Medicine Research Array (Thermo Fisher Scientific, Waltham, MA, USA). After hybridization, the nonspecific background signals were removed by washing under stringent conditions. Genotyping was performed using a Theragen PMRA array to assess 540,000 SNPs. Quality control measures ensured data accuracy, with a dish quality control > 0.82 and a sample call rate > 0.95.

### 2.4. Imputation and Quality Control

Prephasing was performed using Eagle v2.4.1, and imputation was performed using minimac3 with the 1000 Genomes Project Phase 1 (version 3) East Asian reference haplotype. A total of 5,226,711 SNPs were imputed with r^2^ > 0.8.

### 2.5. Statistical Analysis

Based on the responses to the questionnaire, patch test, and sting test results, a GWAS was conducted for the sensitive and insensitive samples. Logistic regression analysis was performed using age as a covariate. Statistical analyses were performed using PLIKK version 1.9 and IBM^®^ SPSS Statistics v.30 for Windows (https://www.ibm.com/kr-ko/spss, assessed on 1 August 2024). The genome-wide significance was considered at a *p*-value < 5 × 10^−8^, and *p* < 1 × 10^−5^ was set for suggestive association. Manhattan and signal plots were generated in R (version 4.1.2) and LocusZoom (version 0.4.8.2) [20]. Quantitative expression plots were obtained from the GTEX portal (https://www.gtexportal.org/home/ [accessed on 30 August 2024]) to understand the functional importance of the lead SNPs [21].

## 3. Results

### 3.1. Population Characteristics

The comprehensive questionnaire survey, conducted with 1690 participants, revealed SS and NS in 389 and 1301 participants, respectively (Table 1). This survey included questions on general skin status, cosmetic usage, innate skin characteristics, environmental impact, and personal habits. The differentiation between SS and NS was further validated using both patch and sting tests. For SS, the validation criteria were an SLS score ≥ 1 and an average score of stinging and burning sensations ≥ 0.4. In contrast, NS was defined as an SLS score < 1 and an average score of stinging and burning sensations < 0.4. Consequently, 115 SS and 181 NS skin samples were finally selected for GWAS analysis. The questionnaire responses and sting test results significantly differed between the SS and NS groups, with the SS group exhibiting higher sensitivity scores and more pronounced reactions in the sting test than the NS counterparts (Table 2).

### 3.2. Results of GWAS

**Figure 1:**
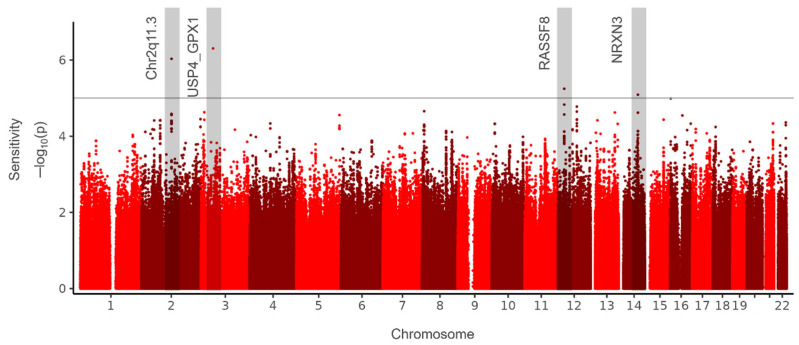
The Manhattan plot illustrates the results of a genome-wide association study associated with the risk of sensitive skin. The horizontal axis of each plot represents the SNP positions across chromosomes 1 to 22, while the vertical axis shows the −log10 transformation of the *p*-values for the association with sensitive skin risk. The solid line on the graphs denotes the genome-wide suggestive threshold (*p* < 1 × 10^−5^). Regions highlighted with gray boxes indicate SNP positions that meet the *p*-value criteria, with the corresponding gene names listed next to each box. SNP, single-nucleotide polymorphism. The different color dots are used to distinguish between the chromosomes.

**Figure 2:**
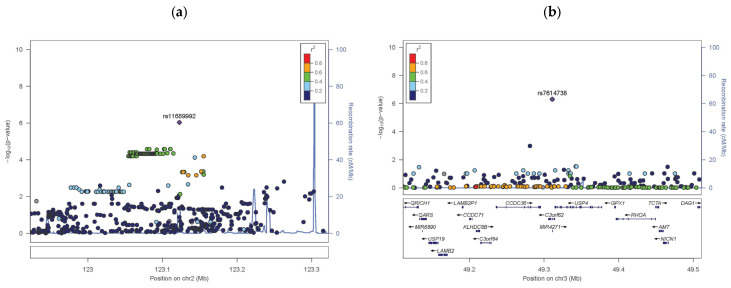

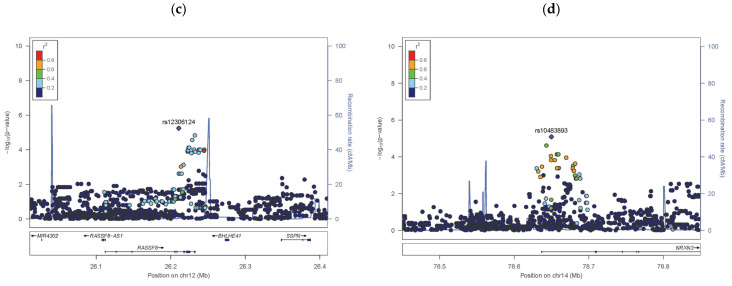
Signal plots that meet the *p*-value criteria indicate SNP regions that were confirmed in the Sensitive Skin GWAS. The lead SNPs were identified by the GWAS and the ±400 kbp region was plotted. The horizontal axis represents the base pair position on the chromosome where the SNPs are located, and the left vertical axis represents the *p*-value transformed into −log10. The vertical axis on the right represents the region where recombination can occur during cell division. SNP, single-nucleotide polymorphism. (**a**) Sensitive: *Chr2q11.3*_rs11689992, (**b**) Sensitive: *USP4*_*GPX1*_rs7614738, (**c**) Sensitive: *RASSF8*_rs12306124, (**d**) Sensitive: *NRXN3*_rs10483893. The arrows indicate the direction of the strand in which each gene is expressed.

GWAS was performed to identify the genetic loci associated with 115 SS and 181 NS samples. GWAS results are illustrated in a Manhattan plot (Figure 1), which displays the distribution of *p*-values across the genome. Although no SNPs met the stringent genome-wide significance threshold (*p* < 5 × 10^−8^) given the limited number of participants with SS, four SNPs exhibited suggestive associations (*p* < 1 × 10^−5^) and are considered to be potential genes associated with SS. Genome construction of these SNP regions reflects signal plots around the major SNPs (Figure 2).

The most notable SNP loci identified in SS includes rs11689992, rs7614738, rs12306124, and rs12306124 (Table 3). The SNP rs11689992, located in the 2q11.3 region of chromosome 2, increased the odds ratio (OR) by 3.67 in SS. This region did not contain any functional genes (Figure 2a). SNP rs7614738 was located at the *USP4* gene locus (Figure 2b). This SNP was identified as an expression quantitative trait locus (eQTL) of *GPX1* in the GTEX portal (Supplementary Figure S1). It increased the odds ratios by 2.34 in SS. *GPX1* plays a role in the management of oxidative stress and is associated with skin sensitivity and inflammation [22]. The SNP rs12306124 was located in the *RASSF8* locus (Figure 2c). Moreover, this SNP was an eQTL for *RASSF8*, as detected using the GTEX portal (Supplementary Figure S2). *RASSF8* is involved in cell growth regulation and apoptosis; its genetic variation may affect skin sensitivity [23]. The rs10483893, located in the *NRXN3* region (Figure 2d), is involved in sensory nerve function [24].

Additionally, we coded the genotypes as follows: homozygous wild allele = 0, heterozygous = 1, and homozygous derived allele = 2. Individual summed scores for cases categorized as slightly, moderately, and severely sensitive were summed and compared. As presented in Supplementary Table S3, we confirmed that a higher score correlates with an increased proportion of moderate and severe cases.

## 4. Discussion

Sensitive skin results from a complex interplay of factors, including impaired skin barrier function, inflammatory responses, and genetic predispositions. However, the precise mechanisms remain unclear. Reduced ceramides weaken the skin’s protective barrier, leading to increased sensitivity [25,26]. Inflammatory mediators such as cytokines and chemokines exacerbate skin sensitivity [27]. Endocrine changes and stress can also alter immune responses, worsening symptoms [28]. Despite advances in understanding these factors, uncertainties persist, particularly regarding the genetic factors and the consistent impact of various external factors. The present study significantly contributes to a growing body of research illustrating the genetic basis of skin sensitivity in the Korean population. We identified four candidate genetic regions—rs11689992 in 2q11.3, rs7614738 in *USP4*, rs12306124 in *RASSF8*, and rs10483893 in *NRXN3*—associated with SS.

*GPX1* plays a key role in managing oxidative stress and is associated with skin sensitivity and inflammation. *RASSF8* affects cell growth and apoptosis, with genetic variations impacting skin sensitivity. *NRXN3* is crucial for sensory nerve function, further influencing skin sensitivity. SNP rs7614738, located in the *USP4* gene locus, was identified as an eQTL for *GPX1* in our study. *USP4* encodes ubiquitin-specific peptidase 4, which regulates intracellular protein degradation via ubiquitination. *USP4* is involved in various cellular processes, including the regulation of the protein expression associated with cell differentiation, such as the expression of SMAD4. It also plays a role in the immune response by influencing the expression of proteins such as IRF4 and IRF8. *USP4* is involved in various signaling pathways, including ARF-BP1 associated with P53, TGF beta receptor 1 involved in TRF-beta signaling, and components of the Wnt signaling such as Beta-Catenin and TCF4 related. In addition, it affects the NF-kB signaling pathway, which is involved in hyaluronan synthesis, through components such as TRAF2 and HAS2 [29]. Notably, *USP4* regulates the degradation of these proteins via ubiquitination [29]. Although no direct research on the skin has been reported, *USP4* is potentially involved in the inflammatory mechanisms associated with chronic metabolic diseases, such as hepatitis and diabetes [30]. Therefore, we suggest a potential relevance to skin sensitivity. Using the GTEx portal site, we confirmed that the SNP is associated with the regulation of the expression of *GPX1*, an antioxidant enzyme gene, indicating a potential link between the SNP and skin sensitivity. Notably, *GPX1* is crucial for the management of oxidative stress, a process linked to skin aging and sensitivity [22]. Upregulated *GPX1* expression is associated with reduced inflammation and improved wound healing [31]. The association between the rs7614738 polymorphism and skin sensitivity underscores the importance of oxidative stress regulation in skin health.

In addition, *RASSF8* encodes a member of the Ras-association domain family of tumor suppressor proteins. The eQTL analysis of the GTEx portal suggested that variations in *RASSF8* potentially influence cellular responses in the skin and affect sensitivity [23]. Moreover, *RASSF8* exhibits tumor-suppressive properties and is involved in cell junction maintenance, which may be related to skin sensitivity through altered cell–cell interactions [32]. SNP rs10483893 in the *NRXN3* gene region encodes neurexin 3, which plays a pivotal role in synaptic function and neural development. Its involvement in skin sensitivity is possibly related to the interactions between sensory nerves and skin cells [24]. The identification of these genetic variants provides valuable insights into the genetic factors correlated with skin sensitivity. These findings may contribute to the development of personalized treatment approaches and cosmetic products tailored to the genetic profiles of individuals. Instead of relying on conventional methodologies, such as questionnaires and various objective and subjective assessments to determine sensitive skin, non-invasive swab-based genomic analysis offers a rapid and precise approach to evaluating and managing SS. Furthermore, targeting suitable specific genetic pathways may effectively mitigate the symptoms and improve skin health.

However, this study has several limitations. For example, the small sample size may have limited the ability to detect SNPs that met the stringent genome-wide significance threshold. Despite the lack of epidemiological investigations on ethnic stratification, hormonal changes, smoking, and environmental changes, we included age as a covariate, as it is the most significant confounding factor accumulatively affected by environmental influences. Moreover, the identified genetic variants may not fully explain the complexity of SS, as skin sensitivity is influenced by both genetic and environmental factors. Further research including larger sample sizes and diverse populations can validate these facts and explore the functional implications of the identified SNPs. In addition, the genetic architecture for skin diseases, such as acne, rosacea, eczema, and psoriasis, varies based on disease-specific characteristics [33]. However, the pathophysiology of sensitive skin remains unclear, and its genetic architecture may vary depending on its definition. This study suggests that certain SNPs related to oxidative stress and neural responses may serve as significant new markers for sensitive skin.

In conclusion, this study provides a foundation for understanding the genetic basis of skin sensitivity in Koreans and reflects the potential directions for future research. By elucidating the genetic factors involved, we can advance our understanding of skin sensitivity and develop targeted interventions to improve the QoL.

## Figures and Tables

**Table 1:** Summary of initial participants with the classification of sensitive skin and non-sensitive skin based on the response to the questionnaire.

		Number of Participants	Mean Age (SD)
Overall		1690	35.3 (9.2)
Non-sensitive		1301	35.8 (8.9)
Sensitive	All	389	34.7 (9.0)
	Slightly	245	34.1 (9.1)
	Moderate	100	36.2 (8.9)
	Severe	44	34.9 (8.2)

**Table 2:** Demographics and characteristics of the target population.

		Non-Sensitive	Sensitive				*p*-Value
			All	Slightly	Moderately	Severely	
Sample size (*n*)		181	115	86	18	11	-
Mean age		34.5 (6.8)	34.5 (7.9)	32.8 (8.3)	35.2 (7.0)	36.3 (6.3)	-
Category A	General skin status	6.6 (1.2)	9.1 (1.5)	8.6 (1.2)	10.1 (1.1)	11.7 (0.6)	<0.001
Category B	Cosmetic application and skin alternation	5.7 (3.8)	21.0 (5.4)	18.7 (3.3)	25.9 (4.1)	30.9 (2.8)	<0.001
Cosmetic application and skin alternation	Cosmetic uses	1.3 (3.8)	6.5 (2.7)	5.7 (2.3)	7.7 (2.4)	10.1 (2.1)	<0.001
	Innate skin characteristics	1.2 (1.5)	4.4 (2.1)	3.6 (1.5)	5.8 (1.9)	7.6 (1.8)	<0.001
	Environmental Skin Changes	1.5 (1.6)	5.8 (1.9)	5.2 (1.6)	7.3 (1.2)	8.3 (0.9)	<0.001
	Living Habits	1.8 (1.2)	4.4 (1.5)	4.1 (1.5)	5.1 (1.4)	4.9 (1.6)	<0.001
Sting test	Mean score of stinging and burning sensations	0.05 (0.07)	0.70 (0.34)	0.68 (0.33)	0.80 (0.40)	0.66 (0.23)	<0.001

( ) Standard deviations.

**Table 3:** Top lead SNPs of each significant locus.

SNP	Chr:Bp	Mapped	eQTL Genes	Effect	Effect Allele Frequency				HWE	Additive Mode	
		Gene	(GTEx Portal)	Allele	This Study	EAS	EUR	AMR	*p*-Value	OR (95% CI)	*p*-Value
rs11689992	2:122,364,982	*chr2q11.3*		A	0.14	0.1	0.43	0.28	1.00	3.67 (2.18–6.16)	9.30 × 10^−7^
rs7614738	3:49,273,698	*USP4*	*GPX1*	C	0.23	0.06	0.48	0.37	0.78	2.34 (1.68–3.27)	4.96 × 10^−7^
rs12306124	12:26,057,827	*RASSF8*	RASSF8	C	0.22	0.22	0.19	0.20	0.23	2.60 (1.72–3.93)	5.68 × 10^−6^
rs10483893	14:78,183,426	*NRXN3*	-	A	0.17	0.23	0.42	0.24	0.21	2.93 (1.83–4.71)	8.15 × 10^−6^

Note. SNP, single-nucleotide polymorphism; Chr, chromosome; Bp, base pair; eQTL, expression quantitative trait locus; EAS, East Asian; EUR, European; AMR, American; Hardy-Weinberg Equilibrium test; OR, odds ratio; CI: Confidence Interval.

## Data Availability

The data that support the findings of this study are not openly available due to reasons of sensitivity and are available from the corresponding author upon reasonable request.

## Supplementary Materials

The following supporting information can be downloaded at: https://www.mdpi.com/article/10.3390/life14111352/s1, Table S1: Diagnostic questionnaire and judgment for assessing self-declared sensitive skin; Table S2: Lead SNPs of sensitive skin GWAS using three genetic modes (additive, dominant, and recessive); Table S3: Distribution of risk allele numbers for each SS grade; Figure S1: GTEX Portal Search for the rs7614738 eQTL for GPX1 gene: Skin-sun exposure and Skin-non-sun exposure tissues showed significantly lower expression for the G allele; Figure S2: GTEX Portal Search for the rs12306124 eQTL for RASSF8 gene: Skin-sun exposed tissues showed higher expression tendency for the C allele.

## Appendix A. Skin Sensitivity Questionnaire

**Question Items**	**Answers (Check the Box)**	
**Self-Reported Sensitive Skin**		
**Do you think your skin is sensitive?**	No □	Yes □
**General Skin Status**		
**1. What is the thickness of your skin?**		
	**1**: Thin	□
	**2**: Normal	□
	**3**: Thick	□
**2. How does your skin typically change?**		
	**1**: Rarely changes	□
	**2**: Occasionally changes	□
	**3**: Frequently changes	□
**3. Do you experience skin issues caused by cosmetics?**		
	**1**: Rarely or Never	□
	**2**: Sometimes	□
	**3**: Often	□
**4. Do you have any skin allergies?**		
	**1**: No	□
	**2**: Yes, occasionally	□
	**3**: Yes, frequently	□
**5. Do you easily become flushed in the sunlight, or have you experienced sunburn or similar inflammation?**		
	**1**: No	□
	**2**: Yes, occasionally	□
	**3**: Yes, frequently	□
**Related to Cosmetic Use**		
1. After I use alcohol-rich cosmetics, my skin appears seriously burnt and red.	No □	Yes □
2. When I apply cosmetics, I experience acne breakouts or skin congestion.	No □	Yes □
3. When I use cosmetics with strong scent, my skin experiences side effects.	No □	Yes □
4. When using cosmetics, I always feel itching, heat. Burning, or other sensations	No □	Yes □
5. I can’t use cosmetics freely.	No □	Yes □
6. I only use the cosmetic products that I often use.	No □	Yes □
7. In the early period of changing cosmetics, I experience mild side effects that disappear with continuous use.	No □	Yes □
8. I only use soaps that I have regularly applied before.	No □	Yes □
9. When I change cosmetics, I experience many skin issues.	No □	Yes □
10. I have never used special cosmetics tailored for sensitive skin.	No □	Yes □
11. When I used special cosmetics tailored for sensitive skin, I experience side effects.	No □	Yes □
12. When using cosmetics, my face has become swollen before.	No □	Yes □
13. Thick makeup can easily cause acne on my face.	No □	Yes □
14. I may experience some skin side effects after massage.	No □	Yes □
15. When removing the facial pack, I might feel red or hot on my face.	No □	Yes □	
**Related to Congenital Traits**		
1. Have atropic skin.	No □	Yes □
2. Had the atropic skin in childhood.	No □	Yes □
3. Somebody in my family has atropine skin.	No □	Yes □
4. My family comprise individuals who can’t use cosmetics freely.	No □	Yes □
5. Skins of all my family members are comparatively sensitive.	No □	Yes □
6. The skin is comparatively thin.	No □	Yes □
7. Blood vessel line are visible on the cheek.	No □	Yes □
8. Face easily turns red and it is difficult to recover after turning red	No □	Yes □
9. Sensitive to metal or jewelry.	No □	Yes □
10. Sensitive to pollen.	No □	Yes □
11. Sensitive to food.	No □	Yes □
12. Suffered from skin measles or dermatitis.	No □	Yes □
13. If something grows on the skin, it is difficult to disappear.	No □	Yes □
14.When I am stung or bitten by insects, the swelling become larger than that of others.	No □	Yes □
**Related to Environments**		
1. If I am exposed to sunlight, my face becomes red, hot, or itches rapidly.	No □	Yes □
2. If I am exposed to cold wind, my face turns red.	No □	Yes □
3. In a dusty area, my face itches and produces unusual substances.	No □	Yes □
4. My skin changes with weather fluctuations.	No □	Yes □
5. Temperature differences cause changes in my facial skin.	No □	Yes □
6. My skin condition is influenced by changes in the surrounding environment.	No □	Yes □
7. My skin may worsen if the water or soil changes.	No □	Yes □
8. My skin seems to change with the seasons.	No □	Yes □
9. If I perspire, my face itches.	No □	Yes □
**Related to Living Habits**		
1. Have coprostasis.	No □	Yes □
2. Often lack sleep.	No □	Yes □
3. If I have pressure, my skin will often become loose or produce acne.	No □	Yes □
4. Feel that my skin alters before or after menstruation.	No □	Yes □
5. After I eat spicy food, my face will produce acne.	No □	Yes □
6. My skin lacks adaptability.	No □	Yes □
7. I will take notice of something growing on my face.	No □	Yes □
8. I feel cool in my hands and feet.	No □	Yes □
9. Often use ointments on my face.	No □	Yes □
10. Because I experienced side effects from using ointments, I cannot use them.	No □	Yes □
This questionnaire is powered by Amorepacific, and all rights are reserved by Amorepacific.		
