# Supplementary material for: Genetic Variants Associated with Sensitive Skin: A Genome-Wide Association Study in Korean Women
Source: Life (Basel). 2024 Oct 22;14(11):1352. doi: 10.3390/life14111352 (PMC11595562; doi:10.3390/life14111352)
Supplement: Supplementary file 1 [file life-14-01352-s001.zip › life-3232629-supplementary.pdf]

Supplementary Information for

Genetic Variants Associated with Sensitive Skin: A Genome-Wide Association Study in Korean Women

Seoyoung Kim [1,2,†], Kyung-Won Hong [3,†], Mihyun Oh [1], Susun An [1], Jieun Han [1], Sodam Park [1], Goun Kim [1] and Jae Youl Cho [2,*]

Supplementary Tables

Supplementary Table S1. Diagnostic questionnaire and judgment for assessing self-declared sensitive skin.

Questionnaire		Total Score	Weighted value
Weighted value of the general skin status (A) (5–15 points)	Experience of skin discomfort after using cosmetics, sun sensitivity and inflammation, allergy, skin change, skin thickness	5–8 points	1
		9–10 points	2
		11–12 points	3
		≥13 points	4
Weighted value of cosmetic application and skin alternation (B) (0–48 points)	Cosmetic uses (15 questions)	<11 points	1
	Innate skin characteristics (14 questions)	<18 points	2
	Environmental skin changes (9 questions)	<26 points	3
	Living Habits (10 questions)	≥26 point s	4

Estimated sensitive skin			
Sensitive skin diagnostic classification (2*(A)+3*(B))	<10	GRADE I	Non-sensitive
	10-14	GRADE II	Slightly sensitive
	15-17	GRADE III	Moderately sensitive
	≥18	GRADE IV	Strongly sensitive

Sensitive skin can be diagnosed using the [2*(A) + 3*(B)] formula, where A is the weighted value of the general skin status and B is a cosmetic application and skin alteration. A diagnostic value ≤9 indicates non-sensitive skin, whereas however, a score >18 indicates strongly sensitive skin.

Supplementary Table S2. Lead SNPs of sensitive skin GWAS using three genetic modes (additive, dominant, and recessive).

SNP	Chr:Bp	Mapped Gene	eQTL Genes (GTEx Portal)	Effect Allele	Effect Allele Frequency				HWE	Additive mode		Dominant mode		Recessive mode	
					This study	EAS	EUR	AMR	p-value	OR (95% CI)	p-value	OR (95% CI)	p-value	OR (95% CI)	p-value
rs11689992	2:122364982	chr2q11.3	-	A	0.14	0.1	0.43	0.28	1.00	3.67 (2.18-6.16)	9.30×10^{-7}	3.66 (2.11-6.34)	4.00×10^{-6}	7.95 (0.91-69.13)	6.0×10^{-2}
rs7614738	3:49273698	*USP4*	*GPX1*	C	0.23	0.06	0.48	0.37	0.78	2.34 (1.68-3.27)	4.96×10^{-7}	2.90 (1.77-4.77)	2.66×10^{-5}	5.27 (2.02-13.76)	6.78×10^{-4}
rs12306124	12:26057827	*RASSF8*	*RASSF8*	C	0.22	0.22	0.19	0.2	0.23	2.60 (1.72-3.93)	5.68×10^{-6}	2.08 (1.28-3.38)	3.06×10^{-3}	3.40 (1.24-9.36)	1.80×10^{-2}
rs10483893	14:78183426	*NRXN3*	-	A	0.17	0.23	0.42	0.24	0.21	2.93 (1.83-4.71)	8.15×10^{-6}	2.93 (1.75-4.91)	4.43×10^{-5}	7.69 (1.63-36.35)	1.00×10^{-2}

Supplementary Table S3. Distribution of risk allele numbers for each SS grade.

Risk Allele No.	Grade [n (%)]				Total [*n*]
	Non-Sensitive	Slightly Sensitive	Moderately Sensitive	Severely Sensitive	
0	61 (85.9%)	8 (11.3%)	2 (2.8%)	0 (0%)	71
1	61 (68.5%)	19 (21.3%)	5 (5.6%)	4 (4.5%)	89
2	48 (63.2%)	20 (26.3%)	6 (7.9%)	2 (2.6%)	76
3	11 (26.8%)	23 (56.1%)	4 (9.8%)	3 (7.3%)	41
4	0 (0%)	11 (84.6%)	0 (0%)	2 (15.4%)	13
5	0 (0%)	3 (75%)	1 (25%)	0 (0%)	4
6	0 (0%)	2 (100%)	0 (0%)	0 (0%)	2

Linear regression analysis for grade using the risk allele number

Odds ratio	CI L95	CI U95	p-value
1.260	1.181	1.346	2.23954E-11

Supplementary Figures

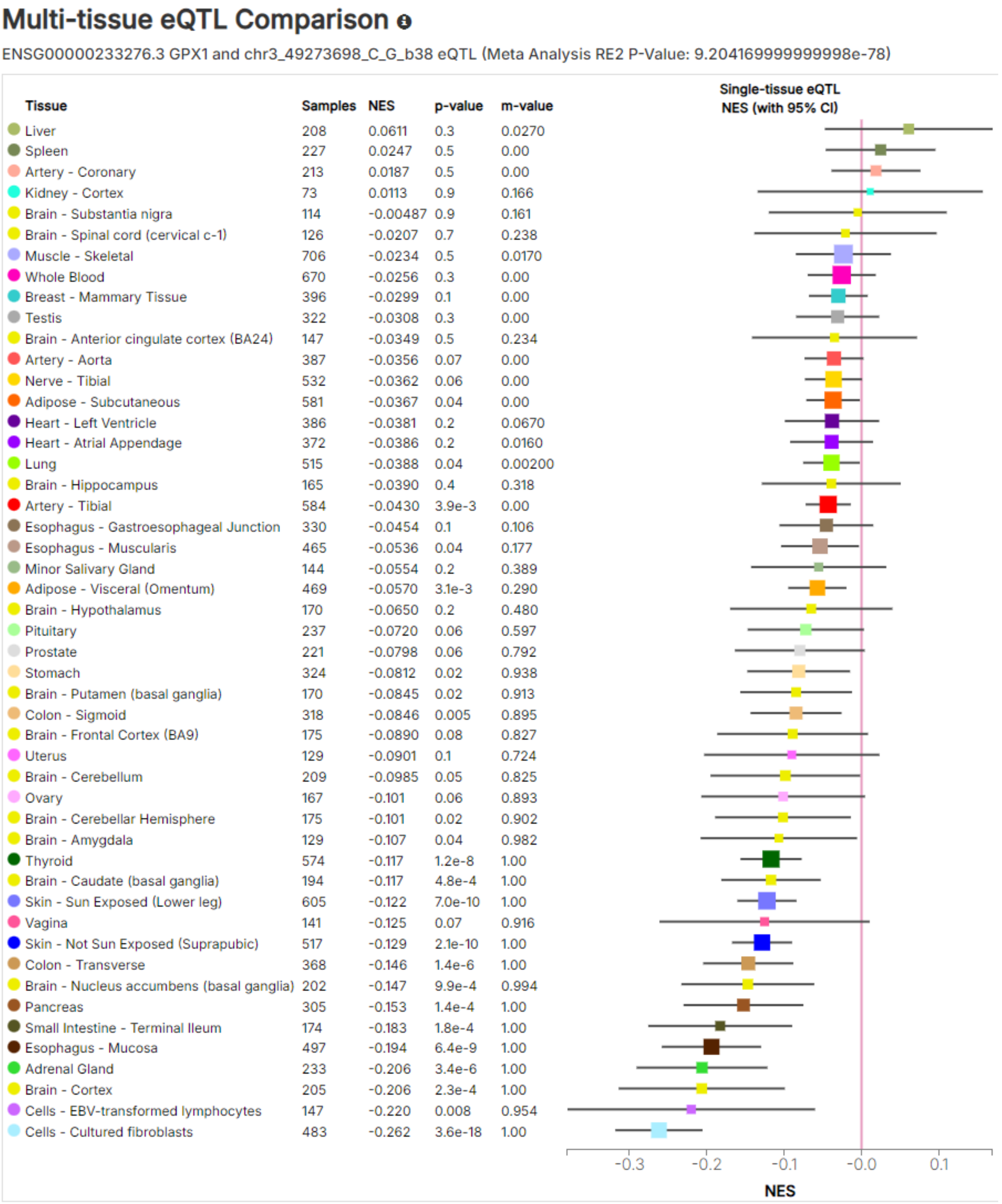

Tissue	Samples	NES	p-value	m-value
Liver	208	0.0611	0.3	0.0270
Spleen	227	0.0247	0.5	0.00
Artery - Coronary	213	0.0187	0.5	0.00
Kidney - Cortex	73	0.0113	0.9	0.166
Brain - Substantia nigra	114	-0.00487	0.9	0.161
Brain - Spinal cord (cervical c-1)	126	-0.0207	0.7	0.238
Muscle - Skeletal	706	-0.0234	0.5	0.0170
Whole Blood	670	-0.0256	0.3	0.00
Breast - Mammary Tissue	396	-0.0299	0.1	0.00
Testis	322	-0.0308	0.3	0.00
Brain - Anterior cingulate cortex (BA24)	147	-0.0349	0.5	0.234
Artery - Aorta	387	-0.0356	0.07	0.00
Nerve - Tibial	532	-0.0362	0.06	0.00
Adipose - Subcutaneous	581	-0.0367	0.04	0.00
Heart - Left Ventricle	386	-0.0381	0.2	0.0670
Heart - Atrial Appendage	372	-0.0386	0.2	0.0160
Lung	515	-0.0388	0.04	0.00200
Brain - Hippocampus	165	-0.0390	0.4	0.318
Artery - Tibial	584	-0.0430	3.9e-3	0.00
Esophagus - Gastroesophageal Junction	330	-0.0454	0.1	0.106
Esophagus - Muscularis	465	-0.0536	0.04	0.177
Minor Salivary Gland	144	-0.0554	0.2	0.389
Adipose - Visceral (Omentum)	469	-0.0570	3.1e-3	0.290
Brain - Hypothalamus	170	-0.0650	0.2	0.480
Pituitary	237	-0.0720	0.06	0.597
Prostate	221	-0.0798	0.06	0.792
Stomach	324	-0.0812	0.02	0.938
Brain - Putamen (basal ganglia)	170	-0.0845	0.02	0.913
Colon - Sigmoid	318	-0.0846	0.005	0.895
Brain - Frontal Cortex (BA9)	175	-0.0890	0.08	0.827
Uterus	129	-0.0901	0.1	0.724
Brain - Cerebellum	209	-0.0985	0.05	0.825
Ovary	167	-0.101	0.06	0.893
Brain - Cerebellar Hemisphere	175	-0.101	0.02	0.902
Brain - Amygdala	129	-0.107	0.04	0.982
Thyroid	574	-0.117	1.2e-8	1.00
Brain - Caudate (basal ganglia)	194	-0.117	4.8e-4	1.00
Skin - Sun Exposed (Lower leg)	605	-0.122	7.0e-10	1.00
Vagina	141	-0.125	0.07	0.916
Skin - Not Sun Exposed (Suprapubic)	517	-0.129	2.1e-10	1.00
Colon - Transverse	368	-0.146	1.4e-6	1.00
Brain - Nucleus accumbens (basal ganglia)	202	-0.147	9.9e-4	0.994
Pancreas	305	-0.153	1.4e-4	1.00
Small Intestine - Terminal Ileum	174	-0.183	1.8e-4	1.00
Esophagus - Mucosa	497	-0.194	6.4e-9	1.00
Adrenal Gland	233	-0.206	3.4e-6	1.00
Brain - Cortex	205	-0.206	2.3e-4	1.00
Cells - EBV-transformed lymphocytes	147	-0.220	0.008	0.954
Cells - Cultured fibroblasts	483	-0.262	3.6e-18	1.00

Supplementary Figure S1. GTEX Portal Search for the rs7614738 eQTL for *GPX1*. Sun-exposed skin tissues exhibited a significantly lower expression of the G allele.

Multi-tissue eQTL Comparison

ENSG00000123094.15 RASSF8 and chr12_26057827_G_C_b38 eQTL (Meta Analysis RE2 P-Value: 0.0000192296)

Tissue	Samples	NES	p-value	m-value
Muscle - Skeletal	706	0.132	3.5e-6	0.308
Pituitary	237	0.0762	0.2	0.0570
Adrenal Gland	233	0.0715	0.3	0.170
Thyroid	574	0.0688	0.02	0.0900
Brain - Hypothalamus	170	0.0588	0.4	0.0950
Heart - Atrial Appendage	372	0.0481	0.2	0.0860
Pancreas	305	0.0435	0.3	0.0860
Brain - Frontal Cortex (BA9)	175	0.0433	0.3	0.0160
Skin - Sun Exposed (Lower leg)	605	0.0423	0.1	0.0290
Brain - Hippocampus	165	0.0337	0.6	0.111
Cells - Cultured fibroblasts	483	0.0304	0.5	0.108
Brain - Nucleus accumbens (basal ganglia)	202	0.0265	0.5	0.0220
Artery - Coronary	213	0.0238	0.7	0.117
Lung	515	0.0231	0.4	0.0170
Esophagus - Mucosa	497	0.0190	0.4	0.0240
Brain - Cortex	205	0.0183	0.7	0.0500
Brain - Spinal cord (cervical c-1)	126	0.0109	0.9	0.153
Brain - Cerebellum	209	0.00807	0.9	0.117
Vagina	141	0.00743	0.9	0.0710
Spleen	227	0.00712	0.9	0.0580
Skin - Not Sun Exposed (Suprapubic)	517	0.00692	0.8	0.0230
Adipose - Visceral (Omentum)	469	0.00574	0.8	0.0200
Brain - Substantia nigra	114	0.000261	1	0.144
Whole Blood	670	-	-	-
Cells - EBV-transformed lymphocytes	147	-	-	-
Brain - Anterior cingulate cortex (BA24)	147	-0.0002571		0.131
Artery - Tibial	584	-0.00784	0.8	0.0300
Breast - Mammary Tissue	396	-0.0106	0.8	0.0560
Heart - Left Ventricle	386	-0.0116	0.8	0.0670
Liver	208	-0.0195	0.8	0.192
Stomach	324	-0.0197	0.5	0.0860
Artery - Aorta	387	-0.0205	0.5	0.0550
Minor Salivary Gland	144	-0.0222	0.7	0.133
Colon - Transverse	368	-0.0228	0.3	0.0590
Prostate	221	-0.0251	0.6	0.128
Adipose - Subcutaneous	581	-0.0385	0.2	0.143
Brain - Putamen (basal ganglia)	170	-0.0392	0.3	0.130
Nerve - Tibial	532	-0.0442	0.1	0.208
Kidney - Cortex	73	-0.0485	0.7	0.223
Brain - Amygdala	129	-0.0675	0.5	0.213
Colon - Sigmoid	318	-0.0785	0.08	0.386
Brain - Cerebellar Hemisphere	175	-0.0864	0.1	0.385
Brain - Caudate (basal ganglia)	194	-0.0874	0.06	0.328
Testis	322	-0.0902	0.005	0.619
Small Intestine - Terminal Ileum	174	-0.0950	0.01	0.523
Uterus	129	-0.0965	0.4	0.213
Esophagus - Muscularis	465	-0.109	2.5e-3	0.632
Ovary	167	-0.133	0.1	0.278
Esophagus - Gastroesophageal Junction	330	-0.154	2.4e-3	0.587

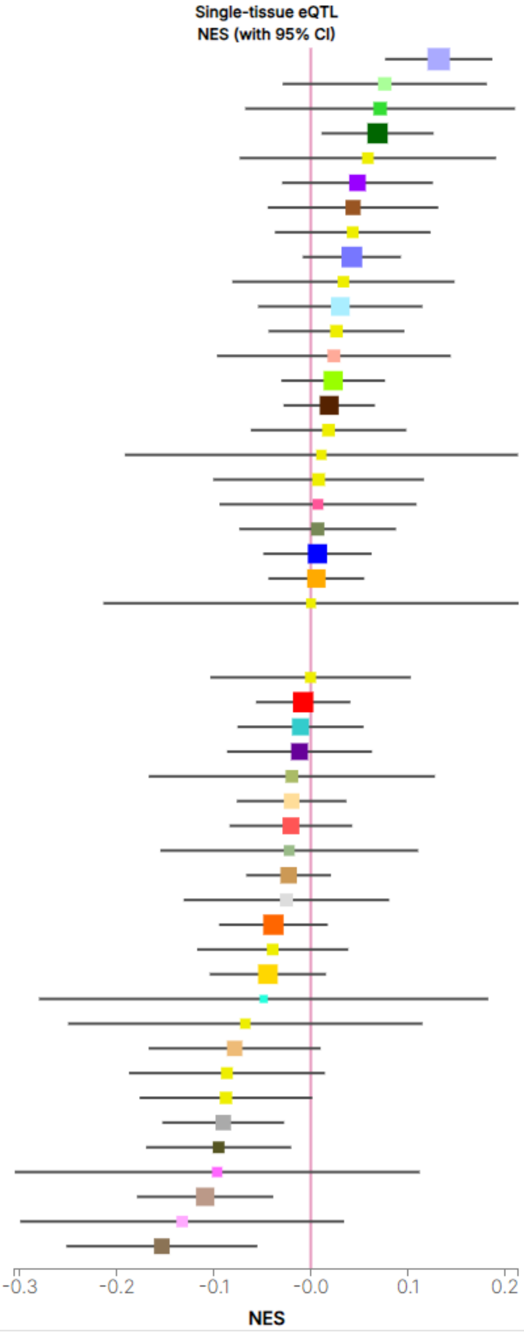

Supplementary Figure S2. GTEX portal search for the rs12306124 eQTL for *RASSF8*. Sun-exposed skin tissues exhibited a higher expression tendency for the C allele.
